# Beyond Surgical Access: Evidence Supporting a Multidimensional Concept of Surgical Invasiveness in Contemporary Cardiac Surgery

**DOI:** 10.3390/jcdd13070315

**Published:** 2026-07-08

**Authors:** Salvatore Poddi, Alessio Rungatscher

**Affiliations:** Division of Cardiac Surgery, University of Verona Medical Center, 37126 Verona, Italy; salvatore.poddi@univr.it

**Keywords:** minimally invasive cardiac surgery, surgical invasiveness, robotic cardiac surgery, post-operative outcomes

## Abstract

Minimally Invasive Cardiac Surgery (MICS) has traditionally been defined according to the extent of surgical access, primarily focusing on the avoidance of full sternotomy and the reduction in incision size. However, the rapid evolution of cardiac surgery, including technological innovation, robotic platforms, hybrid procedures, and enhanced perioperative management, has progressively challenged the adequacy of purely anatomical definitions of invasiveness. Contemporary surgical practice suggests that the overall impact of a procedure on the patient extends beyond the surgical incision itself and includes several physiological and patient-centered dimensions. This narrative review discusses the contemporary meaning of invasiveness in cardiac surgery and examines the limitations of conventional definitions of MICS based exclusively on surgical exposure. This narrative review is based on a non-systematic literature search of PubMed, Scopus, and Web of Science, and uses a thematic synthesis approach to explore the multidimensional concept of surgical invasiveness in cardiac surgery. Particular attention is given to the growing role of patient-centered outcomes and perioperative burden in defining procedural invasiveness. Building upon emerging conceptual perspectives in the literature, this review highlights a multidimensional interpretation of MICS, in which technical, physiological, and recovery-related factors collectively contribute to the assessment of surgical invasiveness.

## 1. Introduction

Minimally Invasive Cardiac Surgery (MICS) has progressively evolved from a technical alternative to full sternotomy into a broader surgical approach aimed at reducing procedural trauma while preserving safety and efficacy. Despite its widespread adoption and continuous technological progress, a universally accepted definition of MICS is still lacking.

The Society of Thoracic Surgeons defines MICS as “any procedure not performed with full sternotomy and cardiopulmonary bypass (CPB) support”, while the American Heart Association describes it as “a small chest wall incision that does not include the conventional full sternotomy” [1]. Both definitions primarily emphasize surgical access, with incision size and avoidance of sternotomy representing the main criteria.

However, MICS should not be limited to the concept of smaller incisions. As previously highlighted by Chitwood, it represents a “philosophy” of cardiac surgery focused on minimizing the overall impact of the procedure on the patient [2]. In this context, the goal is not only to reduce surgical exposure but to decrease the global procedural burden compared with conventional sternotomy.

More recently, our group has proposed a multidimensional concept of MICS, extending beyond access alone [3]. The present narrative review builds upon that framework by critically appraising and synthesizing the available evidence to assess whether this broader definition is supported by current clinical outcomes and contemporary surgical practice, and to determine whether MICS is truly less invasive or merely represents a reduction in skin incision size.

## 2. Methodology

The format of a narrative review is the most suitable because of the heterogeneous nature of the current literature on this topic. The narrative review is the best tool to provide a complete synopsis and describe the current status. The present review explores the concept of surgical invasiveness in cardiac surgery in the context of MICS. A non-systematic literature search was performed in PubMed, Scopus, and Web of Science to identify relevant studies on MICS definitions, surgical access strategies, cardiopulmonary bypass effects, perioperative outcomes, and patient-reported outcomes. The literature search was performed using the keywords “minimally invasive cardiac surgery”, “totally endoscopic cardiac surgery”, and “robotic cardiac surgery”, in combination with outcome-related terms (e.g., “outcomes” and “results”) and appropriate Boolean operators.

Inclusion criteria comprised review articles, descriptive cohort studies, and case reports relevant to the topics identified through the previously listed queries. Exclusion criteria were duplicates, non-English articles, and studies clearly outside the scope of this review. Articles about percutaneous procedures were excluded as they are non-surgical procedures. No time restrictions were applied in order to include both foundational and recent literature relevant to the conceptual development of the field. Priority was given to peer-reviewed original articles, consensus statements, and key review papers considered relevant to the aim of this study.

The literature was analyzed thematically to identify the main dimensions of surgical invasiveness, including technical, physiological, and patient-centered aspects. As a first step, we read the titles of each study and selected the ones that referred to our topics. As a second step, we read abstracts to select the potentially interesting papers. The latter were carefully and thoroughly read (as a third step) to select the most appropriate articles for inclusion. Articles’ references were reviewed to include accordingly. To avoid bias, the search and selection process was carried out by two authors independently (S.P., A.R.). In case of discrepancies between the two authors regarding study selection or interpretation of the evidence, consensus was reached through discussion. A reflexive approach was adopted by the authors to ensure transparency and awareness of potential interpretive bias. The literature search and selection were performed between March and May 2026. At the end of our search and selection process, we decided to include 23 studies.

## 3. Rethinking Surgical Invasiveness

Surgical invasiveness in cardiac surgery has traditionally been defined by the extent of surgical access, with full sternotomy considered the standard reference approach. Accordingly, MICS has largely been described in terms of reduced incision size or avoidance of sternotomy, as reflected in early consensus definitions [1,4]. However, this perspective captures only one dimension of the overall surgical impact.

Beyond access, invasiveness is also determined by physiological injury, including tissue trauma, CPB-related effects, and systemic inflammatory response. In cardiac surgery, these factors are particularly relevant, as extracorporeal circulation and ischemia–reperfusion injury contribute significantly to the overall procedural burden [5].

Therefore, invasiveness should be considered a composite construct including technical, physiological, and biological dimensions. From this perspective, access-based definitions alone may underestimate relevant determinants of recovery, including organ dysfunction and postoperative functional outcomes. This supports a shift toward a multidimensional concept of invasiveness that better reflects the complexity of modern cardiac surgical care.

## 4. From Technical Access to Global Surgical Burden and Patient Impact

Although reduced incisions and avoidance of full sternotomy remain central features of minimally invasive approaches, perioperative burden is also influenced by systemic physiological stress and postoperative recovery dynamics.

Among these factors, CPB represents a major contributor to the physiological burden of cardiac surgery. Contact between blood components and extracorporeal circuit surfaces, together with ischemia–reperfusion mechanisms, activates inflammatory and coagulation pathways that may lead to endothelial dysfunction, capillary leakage, and transient organ impairment [6,7]. Despite substantial technological advances in perfusion systems and perioperative management, CPB-related inflammation continues to influence postoperative outcomes, particularly in high-risk or frail patients. In recent years, increasing attention has been directed toward strategies aimed at reducing the inflammatory response associated with extracorporeal circulation [8]. Notably, minimally invasive surgical access does not necessarily imply reduced exposure to CPB or its associated physiological effects. Consequently, the extent of surgical access and the physiological burden related to extracorporeal circulation should be regarded as complementary, yet distinct, determinants of procedural invasiveness.

Importantly, the consequences of cardiac surgery extend beyond perioperative morbidity and mortality. Parameters such as duration of mechanical ventilation, Intensive Care Unit (ICU) and hospital length of stay, postoperative pain, mobilization, and return to baseline activity increasingly represent meaningful indicators of procedural impact. In this context, Enhanced Recovery After Surgery protocols have emphasized the importance of reducing the overall surgical stress response through multidisciplinary perioperative optimization [9]. Interventions such as multimodal analgesia, early extubation, early mobilization, and optimized perioperative nutrition should be regarded as integral strategies for reducing the physiological burden of cardiac surgery, complementing rather than replacing the benefits derived from minimally invasive surgical access.

At the same time, patient-reported outcome measures have become increasingly relevant in the assessment of cardiac surgical outcomes. Parameters such as quality of life, physical functioning, pain perception, and return to normal daily activities may provide clinically meaningful information that is not fully captured by conventional morbidity and mortality endpoints. Recent longitudinal analyses comparing robotic mitral valve repair and conventional sternotomy demonstrated excellent postoperative quality-of-life recovery in both groups, highlighting the importance of integrating patient-centered recovery metrics into the evaluation of surgical impact [10].

Overall, current evidence suggests that the burden of cardiac surgery should be interpreted through the integration of technical, physiological, and patient-centered dimensions. Within this perspective, surgical access represents only one element contributing to the overall impact of the procedure on the patient.

## 5. Toward a Contemporary Definition of MICS

### 5.1. From Access-Based Surgery to Multidimensional Invasiveness

The evolution of MICS has progressively challenged the traditional notion that surgical invasiveness is primarily determined by the extent of surgical access. Historically, definitions of MICS proposed by major scientific societies have focused predominantly on access-based criteria, emphasizing avoidance of full sternotomy or reduction in thoracic incision as the primary defining feature [1]. While these definitions were essential in standardizing early terminology, they provide only a structural description of the procedure and do not capture its functional or physiological consequences.

Accordingly, limiting the definition of MICS to the characteristics of surgical access risks overlooking important determinants of overall invasiveness, including peri- and post-operative factors. A more comprehensive definition should therefore reflect both the anatomical and physiological dimensions of surgical invasiveness.

### 5.2. Clinical Evidence of Reduced Perioperative Burden

Clinical evidence accumulated over the past decades suggests that minimally invasive and robotic cardiac surgery are associated with measurable improvements in perioperative outcomes beyond the operating field. In mitral valve surgery, multiple large institutional and propensity-matched analyses have demonstrated that minimally invasive approaches are associated with shorter duration of mechanical ventilation, reduced ICU and hospital length of stay, lower transfusion requirements, and reduced perioperative morbidity compared with conventional sternotomy [11,12,13,14,15,16].

Suri et al. reported in a large series of robotic mitral valve repair that minimally invasive approaches can achieve excellent surgical outcomes while maintaining low perioperative morbidity and facilitating rapid postoperative recovery [11]. Similarly, Mihaljevic et al. demonstrated that robotic mitral valve repair is associated with shorter hospitalization and faster early recovery compared with conventional sternotomy, without compromising repair durability or safety [12].

These findings have been reinforced by comparative analyses including propensity-matched cohorts, in which minimally invasive and robotic mitral surgery were associated with significantly reduced resource utilization, including shorter ICU and hospital stays and lower transfusion requirements [13]. Hawkins et al. further confirmed that less invasive approaches are associated with improved early postoperative recovery profiles and reduced perioperative morbidity when compared with conventional sternotomy approaches [13].

In addition, Goldstone et al. demonstrated in a propensity-matched analysis that minimally invasive mitral valve surgery provides at least equivalent operative outcomes while reducing perioperative resource utilization, including blood transfusion requirements and hospitalization duration [14].

Beyond mitral valve surgery, minimally invasive approaches have also demonstrated excellent outcomes in aortic valve replacement (AVR) and coronary artery bypass grafting (CABG). Lamelas reported excellent results with right anterior minithoracotomy AVR, whereas Zoni demonstrated favorable outcomes using a totally endoscopic technique [17,18]. Moreover, other authors showed that robotic-assisted AVR and CABG have shown good clinical results, further confirming the safety and efficacy of MICS [19,20,21].

These consistent findings across multiple high-volume centers support the concept that MICS can be associated with a true reduction in early postoperative burden rather than merely a cosmetic or access-related modification. Nevertheless, the available evidence is largely limited to short- and mid-term follow-up, whereas robust long-term data remain scarce. This is particularly relevant in the field of valvular surgery, where extended follow-up is essential to assess valve durability, freedom from valve-related complications and reinterventions, and the long-term impact of minimally invasive approaches on clinical outcomes.

Despite its numerous advantages, MICS also presents some challenges. The adoption of minimally invasive techniques is associated with a significant learning curve, and longer operative times may be observed, especially during the early stages of a surgeon’s experience [22,23]. As proficiency increases, these limitations generally become less pronounced [22]. Also, MICS is currently applicable to selected procedural and patient subsets and is therefore not universally transferable to all cardiac surgical scenarios; in specific complex cases, conventional sternotomy remains the preferred approach due to technical and physiological considerations, including myocardial protection and operative times.

### 5.3. From Clinical Outcomes to Patient Experience

From a clinical perspective, the observed improvements in early recovery following MICS have been consistently reported in large single-center experiences. In the Cleveland Clinic series of over 1000 robotic mitral valve repairs, excellent procedural safety was demonstrated together with favorable early postoperative outcomes, including short ICU and hospital length of stay and low perioperative morbidity [22]. These findings support the concept that less invasive surgical approaches are associated with an improved early recovery profile compared with conventional sternotomy techniques. However, it is important to emphasize that most clinical studies assess surrogate endpoints such as ventilation time, ICU stay, and transfusion requirements, rather than direct physiological markers of invasiveness. As such, these parameters should be interpreted as clinically meaningful proxies of overall surgical burden. In parallel, enhanced recovery principles in cardiac surgery have further emphasized the importance of reducing perioperative stress and optimizing recovery pathways. Although not specific to MICS, these protocols highlight the clinical relevance of variables such as early extubation, ICU length of stay, and functional recovery as key indicators of surgical impact [9].

Moreover, patient-centered outcomes have become increasingly relevant in evaluating different surgical strategies. Parameters such as quality of life, functional recovery, pain perception, and return to daily activities provide additional dimensions of outcome assessment that are not fully captured by traditional endpoints alone. Recent longitudinal evidence comparing robotic mitral valve repair and conventional sternotomy has demonstrated excellent and comparable recovery of quality-of-life outcomes across surgical approaches, reinforcing the importance of integrating patient-reported outcomes into the evaluation of surgical invasiveness [10].

### 5.4. From Incision to Impact: A New Definition of MICS

Taken together, the available evidence suggests that MICS may be associated with consistent reductions in perioperative resource utilization, postoperative complications, and recovery time. These findings indicate that the impact of MICS may extend beyond surgical access and reflect a broader reduction in the overall burden of cardiac surgery.

Currently available definitions of MICS are predominantly access-based and do not incorporate a formal multidimensional framework [1]. This limitation underlines the need for broader conceptual models integrating technical, physiological, and recovery-related dimensions.

**Figure 1:**
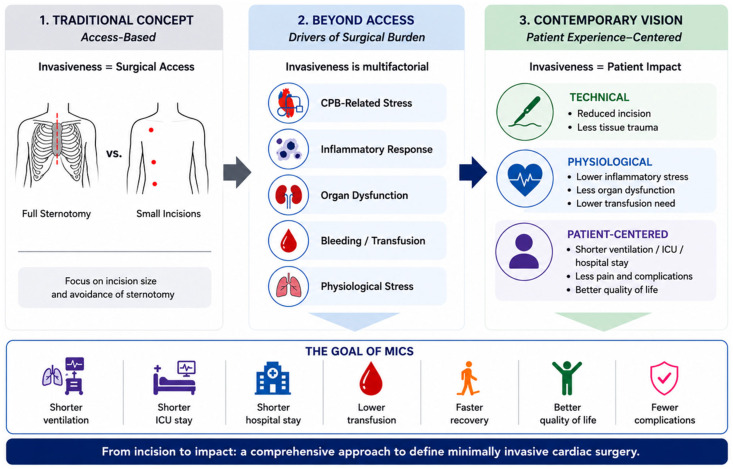
Paradigm shift in the definition of minimally invasive cardiac surgery. CPB: Cardiopulmonary Bypass; ICU: Intensive Care Unit.

Within this context, the new concept recently proposed by our group provides a structured and clinically relevant expansion of the definition of MICS (Figure 1). As per our definition, MICS should refer to every cardiac surgery approach and/or technique (so percutaneous procedures are excluded) which allows decreased physical trauma (smaller skin incisions, sternum and/or costal sparing), avoidance or reduction in invasive tools (CPB, cardioplegic arrest, hypothermia, mechanical ventilation, intubation, ICU stay), hospital stay shortening, and prompt return to normal life (that is the “minimally invasive hospital experience” for the patient) without precluding short- and long-term outcomes [3]. This framework incorporates not only surgical access but also key perioperative and recovery-related variables. By integrating these elements, this definition shifts the focus from anatomical access to the global perioperative impact experienced by the patient.

This approach aligns with contemporary evidence showing that reductions in ICU stay, ventilation time, transfusion requirements, and postoperative complications are associated with minimally invasive and robotic cardiac surgery [11,12,13,14,15,16,17,22]. Therefore, this conceptual framework may provide a more functionally relevant and clinically comprehensive definition of MICS compared with traditional access-based definitions.

From a clinical perspective, this multidimensional interpretation may improve the evaluation of surgical strategies by incorporating both technical and recovery-related outcomes. From a research standpoint, it provides a framework for more standardized comparison of surgical approaches, integrating conventional endpoints with perioperative recovery metrics and patient-centered outcomes.

Procedural effectiveness, including the durability of valve repair and replacement, should also be considered when assessing overall surgical invasiveness, although current evidence is largely based on short- to mid-term outcomes, and longer-term studies are needed to fully define its impact.

Future developments in cardiac surgery will likely further strengthen this conceptual transition. Advances in robotic platforms, perfusion technology, perioperative imaging, and artificial intelligence–assisted planning are expected to further reduce procedural trauma and optimize recovery pathways. In parallel, increasing emphasis on enhanced recovery protocols and individualized perioperative care will likely reinforce the importance of functional and patient-centered outcomes in defining surgical success.

Overall, contemporary evidence supports a shift from an access-based definition of MICS toward a multidimensional framework in which surgical invasiveness is defined by its total impact on perioperative physiology and patient recovery. Within this perspective, the concept of a minimally invasive hospital experience may provide a more accurate and clinically meaningful definition of MICS in contemporary cardiac surgery.

The proposed multidimensional framework should be interpreted as a conceptual synthesis rather than a definitive definition and requires further validation across different surgical contexts and outcome datasets.

## 6. Limitations

This narrative review is subject to the inherent limitations of a non-systematic search strategy, which may introduce a degree of selection bias despite the inclusion of key high-quality studies, consensus statements, and landmark literature. In addition, the heterogeneity of surgical techniques, definitions of minimally invasive approaches, and reported outcomes across studies limits the possibility of direct quantitative comparisons, supporting a qualitative synthesis.

The proposed conceptual framework should be interpreted as a hypothesis-generating model that requires future validation in prospective and standardized studies. Nevertheless, by integrating evidence from surgical outcomes, perioperative recovery parameters, and patient-centered measures, this review provides a structured and comprehensive synthesis of the current literature on MICS and its evolving conceptual boundaries. Most available evidence derives from experienced high-volume centers, which may limit generalizability, but also reflects best contemporary practice in the field. Further long-term, multi-center and comparative investigations are needed to better define the clinical implications of this framework.

## 7. Conclusions

MICS has evolved beyond a purely access-based concept, with consistent evidence showing reduced perioperative burden compared with conventional sternotomy, including shorter ICU and hospital stay, lower transfusion requirements, and fewer complications. These data support the notion that MICS may be associated with a real reduction in surgical invasiveness.

Surgical incision alone is therefore insufficient to define the concept of minimal invasiveness, as physiological stress and recovery trajectories are equally relevant in determining the true impact of the procedure.

A multidimensional framework integrating technical, physiological, post-operative, and patient-centered aspects may provide a more appropriate contemporary definition of MICS. This definition may serve as a reference point for future standardization, research, and clinical interpretation of MICS.

## Data Availability

No new data were created or analyzed in this study. Data sharing is not applicable.

## Abbreviations

CPB	Cardiopulmonary Bypass.
ICU	Intensive Care Unit.
MICS	Minimally invasive Cardiac Surgery.

## References

[1] Schmitto J.D., Mokashi S.A., Cohn L.H. (2010). Minimally-invasive valve surgery. J. Am. Coll. Cardiol..

[2] Chitwood W.R. (2022). Historical evolution of robot-assisted cardiac surgery: A 25-year journey. Ann. Cardiothorac. Surg..

[3] Poddi S., Rungatscher A. (2026). Minimally Invasive Cardiac Surgery: A State-of-the-Art Review. J. Clin. Med..

[4] Cohn L.H., Adams D.H., Couper G.S., Bichell D.P., Rosborough D.M., Sears S.P., Aranki S.F. (1997). Minimally invasive cardiac valve surgery improves patient satisfaction while reducing costs of cardiac valve replacement and repair. Ann. Surg..

[5] Bronicki R.A., Hall M. (2016). Cardiopulmonary Bypass-Induced Inflammatory Response: Pathophysiology and Treatment. Pediatr. Crit. Care Med..

[6] Condello I., Santarpino G., Nasso G., Fiore F., Moscarelli M., Mastroroberto P., Speziale G. (2021). Air, inflammation and biocompatibility of the extracorporeal circuits. Perfusion.

[7] Nteliopoulos G., Nikolakopoulou Z., Chow B.H.N., Corless R., Nguyen B., Dimarakis I. (2022). Lung injury following cardiopulmonary bypass: A clinical update. Expert Rev. Cardiovasc. Ther..

[8] Naruka V., Salmasi M.Y., Arjomandi Rad A., Marczin N., Lazopoulos G., Moscarelli M., Casula R., Athanasiou T. (2022). Use of Cytokine Filters During Cardiopulmonary Bypass: Systematic Review and Meta-Analysis. Heart Lung Circ..

[9] Engelman D.T., Ben Ali W., Williams J.B., Perrault L.P., Reddy V.S., Arora R.C., Roselli E.E., Khoynezhad A., Gerdisch M., Levy J.H. (2019). Guidelines for Perioperative Care in Cardiac Surgery: Enhanced Recovery After Surgery Society Recommendations. JAMA Surg..

[10] Bakir N.H., Damara F.A., Burns D.J.P., Houghtaling P.L., DiPaola L.M., Svensson L.G., Blackstone E.H., Malas T., Gillinov M. (2025). Longitudinal quality of life assessment following robotic mitral valve repair versus conventional sternotomy. JTCVS Open.

[11] Suri R.M., Burkhart H.M., Daly R.C., Dearani J.A., Park S.J., Sundt T.M., Li Z., Enriquez-Sarano M., Schaff H.V. (2011). Robotic mitral valve repair for all prolapse subsets using techniques identical to open valvuloplasty: Establishing the benchmark against which percutaneous interventions should be judged. J. Thorac. Cardiovasc. Surg..

[12] Mihaljevic T., Jarrett C.M., Gillinov A.M., Williams S.J., DeVilliers P.A., Stewart W.J., Svensson L.G., Sabik J.F., Blackstone E.H. (2011). Robotic repair of posterior mitral valve prolapse versus conventional approaches: Potential realized. J. Thorac. Cardiovasc. Surg..

[13] Hawkins R.B., Mehaffey J.H., Mullen M.G., Nifong W.L., Chitwood W.R., Katz M.R., Quader M.A., Kiser A.C., Speir A.M., Ailawadi G. (2018). Investigators for the Virginia Cardiac Services Quality Initiative. A propensity matched analysis of robotic, minimally invasive, and conventional mitral valve surgery. Heart.

[14] Goldstone A.B., Atluri P., Szeto W.Y., Trubelja A., Howard J.L., MacArthur J.W., Newcomb C., Donnelly J.P., Kobrin D.M., Sheridan M.A. (2013). Minimally invasive approach provides at least equivalent results for surgical correction of mitral regurgitation: A propensity-matched comparison. J. Thorac. Cardiovasc. Surg..

[15] Arghami A., Jahanian S., Daly R.C., Hemmati P., Lahr B.D., Rowse P.G., Crestanello J.A., Dearani J.A. (2022). Robotic Mitral Valve Repair: A Decade of Experience With Echocardiographic Follow-up. Ann. Thorac. Surg..

[16] Suri R.M., Dearani J.A., Mihaljevic T., Chitwood W.R., Murphy D.A., Trento A., Javadikasgari H., Burkhart H.M., Nifong W.L., Daly R.C. (2016). Mitral valve repair using robotic technology: Safe, effective, and durable. J. Thorac. Cardiovasc. Surg..

[17] Lamelas J., Alnajar A. (2025). Comparing Outcomes of Sternal Sparing Aortic Valve Replacement with and Without Concomitant Ascending Aortic Replacement. Ann. Thorac. Surg..

[18] Zoni D., Cresce G.D., Hinna-Danesi T., Benvegnù L., Poddi S., Gallo M., Sella M., Salvador L. (2023). Endoscopic aortic valve surgery in isolated and concomitant procedures. Interdiscip. Cardiovasc. Thorac. Surg..

[19] Badhwar V., Pereda D., Khaliel F.H., Poffo R., Darehzereshki A., Mehaffey J.H., Yan T.D., Melnitchouk S., Geirsson A., Arghami A. (2024). Outcomes following initial multicenter experience with robotic aortic valve replacement: Defining a path forward. J. Thorac. Cardiovasc. Surg..

[20] Badhwar V., Raikar G.V., Darehzereshki A., Mehaffey J.H., Daggubati R., Wei L.M. (2025). Robotic-Assisted Aortic Valve Replacement and Coronary Artery Bypass Grafting. Ann. Thorac. Surg..

[21] Nisivaco S., Kitahara H., Bhasin R., Patel B., Coleman C., Balkhy H.H. (2025). A decade of robotic beating-heart totally endoscopic coronary bypass (TECAB) at a single institution: Outcomes with 10-year follow-up. J. Thorac. Cardiovasc. Surg..

[22] Gillinov A.M., Mihaljevic T., Javadikasgari H., Suri R.M., Mick S.L., Navia J.L., Desai M.Y., Bonatti J., Khosravi M., Idrees J.J. (2018). Early results of robotically assisted mitral valve surgery: Analysis of the first 1000 cases. J. Thorac. Cardiovasc. Surg..

[23] Nifong L.W., Chitwood W.R., Pappas P.S., Smith C.R., Argenziano M., Starnes V.A., Shah P.M. (2005). Robotic mitral valve surgery: A United States multicenter trial. J. Thorac. Cardiovasc. Surg..

